# Seasonal Variation and Mean Degree of Polymerization of Proanthocyanidin in Leaves and Branches of Rabbiteye Blueberry (*Vaccinium virgatum* Aiton)

**DOI:** 10.3390/plants13131864

**Published:** 2024-07-05

**Authors:** Yasuko Koga, Yuno Setoguchi, Kazuhiro Sugamoto, Yo Goto, Tomonari Hirano, Hisato Kunitake

**Affiliations:** 1 Graduate School of Agriculture, University of Miyazaki, 1-1 Gakuenkibanadainishi, Miyazaki 889-2192, Japan; 2 Interdisciplinary Graduate School of Agriculture and Engineering, University of Miyazaki, 1-1 Gakuenkibanadainishi, Miyazaki 889-2192, Japan; 3 Faculty of Engineering, University of Miyazaki, 1-1 Gakuenkibanadainishi, Miyazaki 889-2192, Japan; 4 Biolabo Co., Ltd., Kobe 650-0047, Japan; 5 Faculty of Agriculture, University of Miyazaki, 1-1 Gakuenkibanadainishi, Miyazaki 889-2192, Japan

**Keywords:** polyphenol, antioxidant capacity, epicatechin, catechin, chlorogenic acid, ascorbic acid

## Abstract

The leaves and branches of rabbiteye blueberry are rich in proanthocyanidins, which are thought to have different physiological activities depending on their structure and degree of polymerization. In this study, we analyzed the constituents of the leaves and branches of rabbiteye blueberry to determine the seasonal variations in polyphenol and proanthocyanidin (PAC) contents as well as their mean degrees of polymerization (mDP). Total PAC content was determined using two methods: The p-dimethylaminocinnamaldehyde (DMACA) method, which measures monomeric PAC, showed an increase from spring to summer in both leaves and branches. On the other hand, using the butanol/HCl method, which measures only polymerized PAC, the PAC content of leaves increased from spring to summer but those of branches remained low throughout the year, showing no significant increase or decrease. Furthermore, analysis of the mDP of PAC showed increases from spring to summer in the leaves of ‘Kunisato 35 gou’. Although the highest value (8.0) was observed in October, values around 4 remained throughout the year in the branches. Since differences in polymerization degree affect absorption in the body and physiological properties such as antioxidant capacity, selecting the appropriate harvest time and plant organs for each purpose is expected to ensure the quality of processed blueberry foods.

## 1. Introduction

Blueberries are shrubby fruit tree crops of North American origin, classified in the genus *Vaccinium* L., section Cyanococcus (Ericaceae). The cultivated species and hybrids in the genus *Vaccinium* are categorized into three main types—highbush blueberry (*V. corymbosum* L.), rabbiteye blueberry (*V. virgatum* Aiton), and rowbush blueberry (*V. angustifolium* Aiton). These shrubs have become a globally important source of fresh fruit while also providing material for processed food industries [1].

Blueberry fruits are rich in phenolic compounds such as anthocyanin, catechin, epictechin, chlorogenic acid, rutin, and proanthocyanidin (PAC). Blueberries have been suggested to prevent cancer and improve lifestyle-related diseases and are used in functional foods and medicines to maintain and improve health [2]. Recently, functional compounds contained in the leaves and branches of blueberries—in additional to those in the fruit—have attracted attention [3,4].

**Figure 1:**
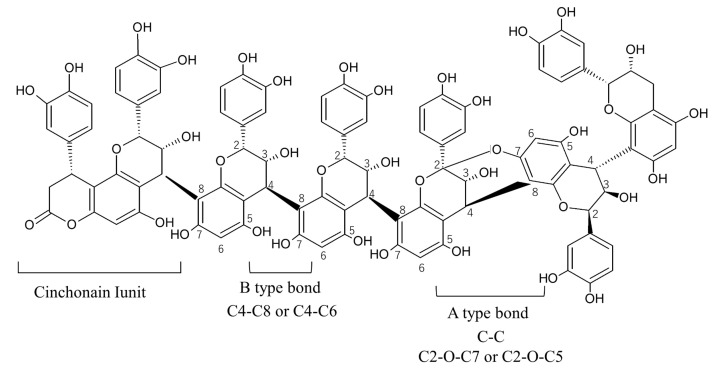
Structural formula of proanthocyanidin. This figure is based on Matsuo et al. (2010) [10].

In 2014, we registered the first specific cultivar for leaves and branches, ‘Kunisato 35 gou’ (No. 21636), which is intercrossed with rabbiteye blueberry ‘Homebell’, with the Japanese Ministry of Agriculture, Forestry and Fisheries [5]. Blueberry leaves contain more phenols and flavonoids than their fruit [6] and have been reported to have antioxidant, anti-fatty liver, antihypertensive, and antidiabetic effects [7,8,9]. In addition, the leaves of ‘Kunisato 35 gou’ contain large amounts of PAC, a type of polyphenol, among its functional components. PAC is composed of a B-type bond, where monomers are interflavan-linked at positions 4 to 8; an A-type bond, where monomers are linked by two interflavan bonds; and a Cinchonain I unit, where a phenylpropane is attached to a monomer (Figure 1) [10]. Furthermore, it is speculated that this component is a characteristic structure polymerized into various sizes. In particular, structural analysis using acid thiolysis revealed that the average degree of polymerization of PAC in purified blueberry leaf extract is very high (7.7) and that epicatechin is the main component [11]. Yamasaki et al. (2021) reported that ‘Kunisato 35 gou’ leaf extract powder inhibited lipid accumulation and uric acid production in adipocytes. They also reported that this activity was higher in the fractions containing catechin, epicatechin, chlorogenic acid, rutin, and low-molecular-weight PAC [12]. In contrast, Sugamoto et al. (2022) reported that blueberry leaf and branch extracts containing highly polymerized PAC had strong inhibitory effects on SARS-CoV-2 replication and were effective in infection protection [13]. Thus, it is suggested that different degrees of PAC polymerization result in different physiological activities. Therefore, a detailed investigation of PAC content and the degree of polymerization in blueberry branches and leaves would provide important data for the effective use of these parts of the plant. However, detailed changes in the polyphenol and PAC contents and their structures in blueberry leaves and branches throughout the year have not yet been determined. In this study, we investigated the seasonal variation of the polyphenol content and composition, PAC content, and the mean degree of polymerization of PAC in blueberry leaves and branches.

While much research has been conducted on blueberry fruits, little is known about their leaves and branches. In particular, there is little information on the branches, which are currently disposed of as industrial waste in Japan. We believe that this research will deepen our knowledge of the components contained in leaves and branches and lead to more effective utilization.

## 2. Results

### 2.1. Seasonal Changes in Leaves and Branches

**Figure 2:**
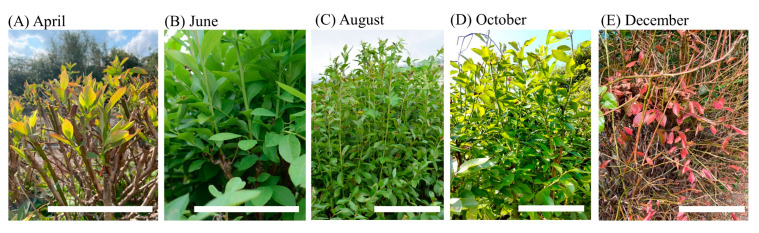
Seasonal changes of leaves and branches of rabbiteye blueberry ‘Kunisato 35 gou’. (**A**): Sprout leaves in April. Bar = 10 cm. (**B**): Mature leaves in June. Bar = 10 cm. (**C**): Mature leaves in August. (**D**): Mature leaves in October. Bar = 10 cm. (**E**): Red coloring of leaves in December. Bar = 15 cm.

Seasonal changes in leaves and branches of rabbiteye blueberry ‘Kunisato 35 gou’ are shown in Figure 2. Leaves began to sprout around March and matured between June and August; they gradually turned red between November and December, with about half of the leaves falling in December. The branches began to grow in March and grew rapidly between June and August; the branch surfaces then turned brown between November and December.

### 2.2. Total Polyphenol Content

**Figure 3:**
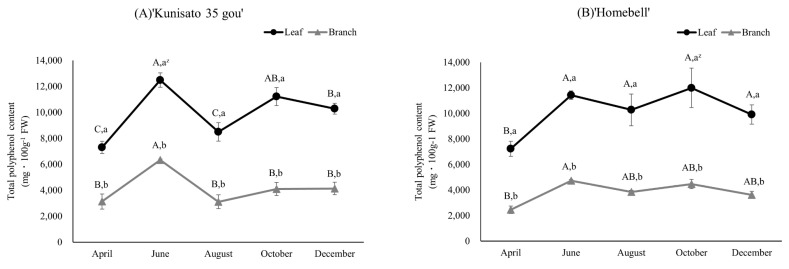
Seasonal changes in the total polyphenol content of the leaves and branches of rabbiteye blueberry ‘Kunisato 35 gou’ (**A**) and ‘Homebell’ (**B**). ^z^ Means followed by different capital letters in the same line (*p* < 0.05) and small letters in the same column (*p* < 0.01) were significantly different based on two-way ANOVA and Tukey’s test (*n* = 3).

The total polyphenol content of ‘Kunisato 35 gou’ and ‘Homebell’ leaves and branches based on the Folin–Ciocalteu reagent method are shown in Figure 3. In both cultivars, the polyphenol content of the leaves was generally higher than that of the branches. The total polyphenol content of the leaves increased between April and June, decreased in August, and then increased again toward December. The highest content of the leaves of ‘Kunisato 35 gou’ and ‘Homebell’ was 12,485.1 and 11,996.9 mg gallic acid equivalent·100 g^−1^ Fresh Weight (FW), respectively. The highest content of the branches of ‘Kunisato 35 gou’ and ‘Homebell’ were 6338.8 and 4733.0 mg gallic acid equivalent·100 g^−1^ FW, respectively.

### 2.3. Polyphenol Component

**Figure 4:**
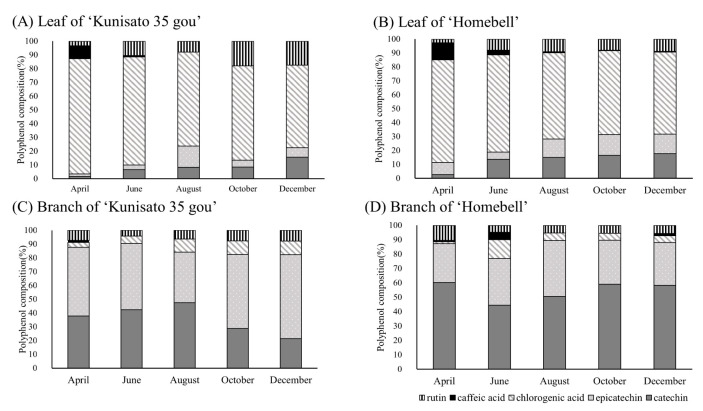
Seasonal changes in the polyphenol composition of leaves and branches of rabbiteye blueberry ‘Kunisato 35 gou’ and ‘Homebell’.

The individual polyphenol compositions of the leaves and branches were analyzed using HPLC (Figure S1) and the results are shown in Figure 4. Five polyphenols, namely catechin, epicatechin, chlorogenic acid, caffeic acid, and rutin, were detected in the leaves and branches of ‘Kunisato 35 gou’ and ‘Homebell’. In both varieties, chlorogenic acid accounted for about 60% to 80% of the total polyphenols in the leaves. In particular, chlorogenic acid accounted for 83.8% of the total polyphenols in the leaves of ‘Kunisato 35 gou’ in April, just after budding. The highest caffeic acid percentages in the leaves of ‘Kunisato 35 gou’ and ‘Homebell’ occurred in April, when they were 9.3% and 12.2%, respectively. They then decreased through winter. The percentage of rutin in ‘Kunisato 35 gou’ leaves increased from 3.4% to 17.5% between April and December. A similar trend was observed in the rutin content of ‘Homebell’ leaves. The percentage of catechins in the leaves of both cultivars tended to increase slowly during the winter. In comparison, catechins and epicatechins accounted for about 80% to 90% of the rutin in the branches of both varieties. The rutin percentage in branches remained approximately 5% to 10% throughout the year. The percentage of chlorogenic acid in the branches of ‘Kunisato 35 gou’ increased gradually from April to December. The percentage of chlorogenic acid in the branches of ‘Homebell’ was the highest (13.0%) in June and decreased during the winter. The percentage of caffeic acid in the branches of both cultivars was low, with the highest value observed in April for ‘Kunisato 35 gou’ and in June for ‘Homebell’, at 1.6% and 5.2%, respectively.

### 2.4. Total PAC Content

**Figure 5:**
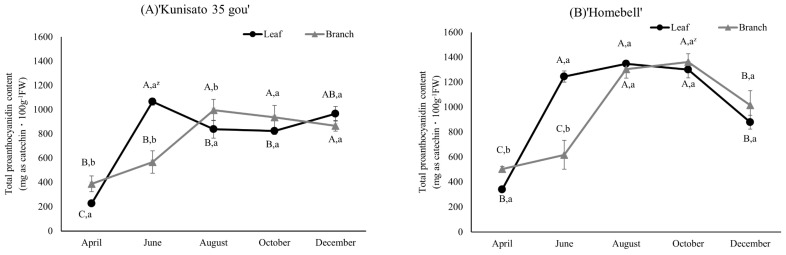
Seasonal changes in the total proanthocyanidin content based on the DMACA analysis method of the leaves and branches of rabbiteye blueberry ‘Kunisato 35 gou’ (**A**) and ‘Homebell’ (**B**). ^z^ Means followed by different capital letters in the same line (*p* < 0.05) and small letters in the same column (*p* < 0.01) were significantly different based on two-way ANOVA and Tukey’s test (*n* = 3).

The total PAC content of rabbiteye blueberry leaves and branches was analyzed using the p-dimethylaminocinnamaldehyde (DMACA) and butanol/HCl methods. First, we analyzed total PACs using the DMACA method. This revealed similar seasonal variations in the leaves and branches of ‘Kunisato 35 gou’ and ‘Homebell’, although there were slight differences in the amounts depending on the season (Figure 5). The total PAC content of ‘Kunisato 35 gou’ leaves increased significantly between April and June, with the highest value observed in June (1064.9 mg catechin equivalent·100 g^−1^ FW). It then decreased in August and slightly increased in December. In comparison, the branches of ‘Kunisato 35 gou’ showed a significant increase between June and August, peaking at 995.6 mg catechin equivalent·100 g^−1^ FW, followed by a gradual decrease during winter, although the difference was not significant.

**Figure 6:**
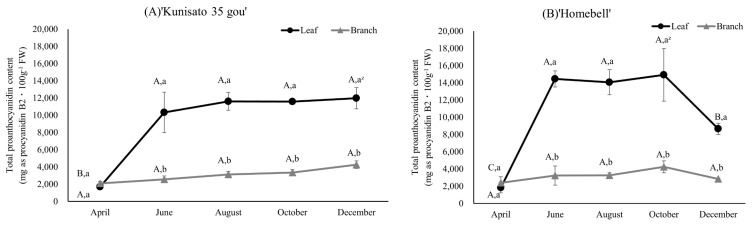
Seasonal changes in the total proanthocyanidin content based on the HCl buthanol analysis method of leaves and branches of rabbiteye blueberry ‘Kunisato 35 gou’ (**A**) and ‘Homebell’ (**B**). ^z^ Means followed by different capital letters in the same line (*p* < 0.05) and small letters in the same column (*p* < 0.01) were significantly different based on two-way ANOVA and Tukey’s test (*n* = 3).

The results of the butanol HCl method for determining the total PAC content of ‘Kunisato 35 gou’ and ‘Homebell’ leaves and branches are shown in Figure 6. In both cultivars, the total PAC content of the leaves was generally higher than the branches. The content of ‘Kunisato 35 gou’ leaves increased significantly between April and June and remained high, peaking at 11,978.1 mg procyanidin B2 equivalent·100 g^−1^ FW in December, although the difference was not statistically significant. On the other hand, the content of the branches showed a different trend from that revealed using the DMACA method, with the branches of both cultivars showing much lower values than leaves from June onward and remaining low all year round in ‘Kunisato 35 gou’.

### 2.5. Mean Degree of Polymerization (mDP) of PAC

**Figure 7:**
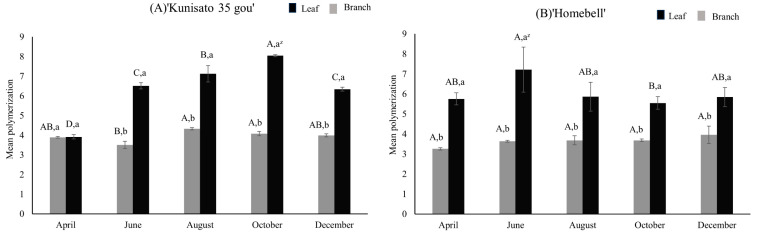
Seasonal changes in the mean polymerization of proanthocyanidin in the leaves and branches of rabbiteye blueberry ‘Kunisato 35 gou’ (**A**) and ‘Homebell’ (**B**). ^z^ Means followed by different capital letters in the same line (*p* < 0.05) and small letters in the same column (*p* < 0.01) were significantly different based on two-way ANOVA and Tukey’s test (*n* = 3).

The mean degree of polymerization (mDP) of PAC in ‘Kunisato 35 gou’ and ‘Home bell’ leaves and branches, as determined using the Q-Exactive mass spectrometry system, are shown in Figure 7. HPLC/MS chromatograms of the proanthocyanidin reaction products are included for reference (Figure S2). In both cultivars, the mDP of the PAC in leaves was generally higher than that of branches, and the mDP of PAC in October, the harvest season, was significantly higher for leaves than branches in both cultivars. The mDP of the PAC in ‘Kunisato 35 gou’ leaves increased between April and October, reaching a significantly higher value (8.0) in October and a significantly lower value in December. The highest value (7.2) was observed for ‘Homebell’ leaves, which increased between April and June and then decreased to maintain a value of approximately 5 to 6. The mDP of the PAC in ‘Kunisato 35 gou’ branches remained at around 4 throughout the year, and that of ‘Homebell’ remained at about 3 to 4 throughout the year.

### 2.6. Antioxidant Activity Analysis

**Figure 8:**
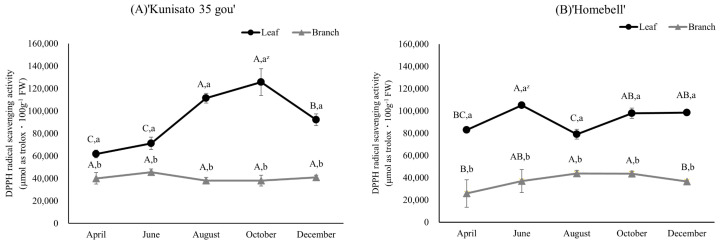
Seasonal changes in the antioxidant activity of leaves and branches of rabbiteye blueberry ‘Kunisato 35 gou’ (**A**) and ‘Homebell’ (**B**). ^z^ Means followed by different capital letters in the same line (*p* < 0.05) and small letters in the same column (*p* < 0.01) were significantly different based on two-way ANOVA and Tukey’s test (*n* = 3).

The 1,1-diphenyl-2-picrylhydrazyl (DPPH) radical scavenging activity results for ‘Kunisato 35 gou’ and ‘Homebell’ leaves and branches are shown in Figure 8; the values are expressed in μmol as Trolox·100 g^−1^ FW. In both cultivars, the leaves showed higher levels of antioxidant activity than the branches at harvest time, in October. In the leaves, the antioxidant activity values of ‘Kunisato 35 gou’ and ‘Homebell’ were 125,695.5 and 105,180.2 μmol as Trolox·100 g^−1^ FW, respectively. In the branches, the antioxidant activity values of ‘Kunisato 35 gou’ and ‘Homebell’ were 45,561.9 and 43,819.8 μmol as Trolox·100 g^−1^ FW, respectively. The antioxidant activity of the leaves of ‘Kunisato 35 gou’ increased significantly between June and October. In both cultivars, the values for the branches did not significantly increase during the course of the year. Differences in the antioxidant activity of rabbiteye blueberry leaves and branches, as well as in the leaves of ‘Kunisato 35 gou’, tended to increase from spring to summer.

## 3. Discussion

Phenylpropanoids in plants, such as lignins, lignans, and norlignans, are secondary compounds that are important for adaptation to various environments. These play important roles in defense against biotic and non-biotic stresses acting on plants, such as viruses, UV rays, and insects. Among phenylpropanoids, PACs have attracted attention as components with many physiological properties [14,15,16]. These compounds are also known to have multifaceted actions, including antibacterial, antioxidant, anti-inflammatory, antidiabetic, anticancer, renoprotective, neuroprotective, cardioprotective, and hepatoprotective effects [17]. Among these phenylpropanoids, PACs, a type of condensed tannin, have attracted attention for their many physiological activities. Recently, PACs in the leaves and branches of rabbiteye blueberry have been reported to exhibit a variety of physiological activities [3,13,18,19]; however, the seasonal variation and mDP of PAC in the leaves and branches of rabbiteye blueberry have not been investigated. In this study, the seasonal variations in PAC and other polyphenols in the leaves and branches of rabbiteye blueberry were determined, and their PAC content and mDP were examined in relation to antioxidant activity.

PAC is a type of polyphenol generally composed of monomers such as catechins and epicatechins. Studies to date have focused on acacia bark extract [20], grape seed extract [21], and pine bark extract [22]. The PAC of blueberry leaves is composed of A-type bonds, where monomers are bonded at C2-O-C7 or C2-O-C5 in addition to C-C bonds between monomers; B-type bonds, where monomers are bonded at C4-C6 or C4-C8; and Cinchonain I units, where phenylpropane is attached to monomers. These are presumably polymerized into various sizes in a characteristic structure [10]. In this study, we used two methods to measure PAC content: The DMACA method was used to measure PAC including monomers, while the butanol/HCl method was used to measure only polymerized PAC [23]. A similar trend in proanthocyanin content was observed in the leaves of both cultivars, increasing between April and June and remaining high through October according to both methods. Using the DMACA method, the branches of both cultivars showed trends similar to those for the leaves, but no significant seasonal variation in proanthocyanin content was observed using the butanol/HCl method. This suggests that the biosynthesis of the monomers catechin and epicatechin is accelerated and that their polymerization proceeds toward summer in the leaves of rabbiteye blueberry, whereas, in the branches, the biosynthesis of monomers is accelerated but the polymerization does not proceed to a high degree. This phenomenon can be inferred from the fact that the mDP of PAC remained at around 4 throughout the year in the branches of ‘Kunisato 35 gou’, whereas in the leaves, it increased between April and October, with a significantly higher value (8.0) observed in October. Bujor et al. (2016) evaluated the seasonal variations in the contents of phenolic compounds and the antioxidant activity of bilberry (*Vaccinium myrtillus* L.) leaves, branches, and fruits collected in May, July, and September for two consecutive years [24]. Their results showed that the seasonal variation was more pronounced in the leaves than in the branches, with higher antioxidant activity and polyphenol content in the leaves in July and September. They also reported that the mDP of PAC was low (2–4) and that 30% of the branches consisted of trimeric compounds composed of A-type and B-type bonds. Rutkowska et al. (2020) also reported that the total PAC content of leaves of rowan (*Sorbus domestica* L.) exhibits a seasonal variation that increases from spring to summer, similar to that observed in rabbiteye blueberry [25]. Since the production and accumulation of secondary metabolites in plants have been reported to be species-specific and strictly responsive to environmental factors associated with seasonal changes [15,26], it will be important to investigate the seasonal variation in PAC biosynthesis and its gene expression.

In the leaves of the medicinal mangrove (*Ceriops tagal*), Zhou et al. (2014) reported a strong positive correlation between the mDP of PAC (around mDP < 10) and antioxidant activity [27]. In the present study, a strong correlation (r^2^ = 0.69) was also observed between the mDP of PAC in ‘Kunisato 35 gou’ leaves and DPPH radical scavenging activity. Many studies have reported that oligomeric PACs exhibit higher antioxidant activity compared to polymeric procyanidins due to the presence of multiple hydroxyl groups [28]. The mDP of PACs may be an important indicator for evaluating the biological activity of plant extracts, along with the total polyphenol content and its composition.

PAC is biosynthesized through a branching flavonoid pathway. The biosynthesis unfolds with a molecule of 4-coumaroyl CoA and three molecules of malonyl-CoA as the starting components. Biosynthesis proceeds with one molecule of 4-coumaroyl CoA and three molecules of malonyl CoA as the starting materials. Leucoanthocyanidin reductase (LAR) and anthocyanidin reductase (ANR) act on leucocyanidins in the flavonoid pathway to produce (+)-catechin and (–)-epicatechin, which are then polymerized into monomeric forms. However, the process underlying this polymerization remains unknown. Recently, Yu et al. (2022) revealed that ascorbic acid (AsA) is a “starter unit” that replaces the flavan-3-ol monomer in the subunit extension of leucocyanidin-derived (+)-catechin. They reported that the oligomerization of PAC does not necessarily proceed via the addition of a single elongation unit [29]. Izumi et al. (1990) reported that the L-ascorbic acid content of satsuma mandarin (*Citrus unshiu* Marc. cv. Hayashi) leaves varied depending on the harvest time, with the highest values being obtained from mature leaves rather than from young leaves [30]. They reported that plants activate AsA synthesis in a light-dependent manner because photosynthesis-induced reactive oxygen species production is enhanced in chloroplasts under light irradiation. AsA increases from spring to summer, when light intensity increases, and it is assumed that this increase promotes the extension of (+)-catechin subunits. In this study, the mean polymerization of PAC in ‘Kunisato 35 gou’ leaves increased from 3.9 to 8.0 between April and October, which is consistent with this hypothesis. We suggest that investigating AsA content may provide clues to the seasonal variation of PAC polymerization.

## 4. Materials and Methods

### 4.1. Plant Materials

The plant material used was 6-year-old rabbiteye blueberry ‘Kunisato 35 gou’, cultivated in the research field of the Faculty of Agriculture, University of Miyazaki (31°49′41.2″ N 131°24′41.0″ E), while 8-year-old rabbiteye blueberry ‘Homebell’ was cultivated as a control. ‘Kunisato 35 gou’ was planted densely using the method of Toyama et al. [5]. ‘Homebell’ was planted at a 3 m spacing for fruit cultivation and grown according to usual methods. The branches and leaves of each cultivar were collected every 2 months starting in April. In addition, samples of each leaf and branch were collected at different times of the year from three independent plants.

The collected branches and leaves were frozen in situ and dried in a freeze-dryer (FDU-2100, EYELA, Tokyo, Japan) and a dry chamber (DRC-1000, EYELA, Tokyo, Japan). These dried samples were ground using a mixer (BUCHI Mixer B-400, BUCHI, Tokyo, Japan) and maintained in a −20 °C freezer.

### 4.2. Polyphenol Analysis

#### 4.2.1. Total Polyphenol Content

Each sample was analyzed for polyphenols by dissolving freeze-dried leaf and branch powder (0.02 g) in 5 mL of 80% (*v*/*v*) methanol, sonicated (US CLEANER, As One, Osaka, Japan) for 15 min, and then filtered through a 0.22 µm membrane filter (Millipore, Bedford, MA, USA). Total polyphenol content was determined according to the partially modified Folin–Ciocalteu reagent method [31]. Briefly, 200 µL of each sample was first mixed with 200 µL of phenol reagent (5.0 mL of distilled water and Folin–Ciocalteu phenol reagent) and 400 µL of saturated sodium carbonate solution. After 30 min, absorbance was read at 760 nm. The standard substance used was gallic acid dissolved in 80% (*v*/*v*) methanol. The total polyphenol content was expressed as g gallic acid equivalents·100 g^−1^ DW. For each treatment, three biological replicates were performed.

#### 4.2.2. Polyphenol Composition Analysis

The polyphenol components of leaves and branches were clarified by using HPLC to separate and identify individual polyphenols. To prepare samples, 0.02 g of frozen leaves and 0.02 g of frozen branches were extracted separately with 5 mL of 80% (*v*/*v*) methanol. The extraction was performed thoroughly at 37 °C for 15 min using an ultrasonic device (US CLEANER, As One, Osaka, Japan). The samples were then filtered through a 0.22 µm membrane filter (Millipore) and immediately subjected to HPLC analysis. The analysis was performed using Inertsil ODS3 (4.6 mm × 250 mm, 5 µm) as the column, and a Prominence LC solution system (Shimadzu Corporation, Kyoto, Japan) was used for the analysis. The chromatographic conditions were as follows: solvent A, 100% (*v*/*v*) ethanol; solvent B, 20 mM potassium phosphate (pH 2.4); column temperature, 40 °C; detection at 280 nm; flow rate, 1.0 mL·min^−1^. The binary gradients were as follows: 85–68% B (0–12 min), 68% B (12–15 min), 68–55% B (15–20 min), 55–85% B (20 min), and 85% (20–29 min). Retention times and spectra were compared with pure standards of chlorogenic acid (FUJIFILM Wako Pure Chemical Corp., Osaka, Japan), catechin (Funakoshi Co., Tokyo, Japan), epicatechin (FUJIFILM Wako Pure Chemical Corp., Osaka, Japan), rutin (FUJIFILM Wako Pure Chemical Corp., Osaka, Japan), and caffeic acid (FUJIFILM Wako Pure Chemical Corp., Osaka, Japan). These extracts were diluted in 100% (*v*/*v*) methanol from 20 to 200 mg·L^−1^. The concentrations were adjusted to obtain similar peak heights so that chromatographic parameters could be easily derived. Finally, the peak areas of the standards and samples were normalized and used for the exact determination of the active ingredient, whose contents were subsequently expressed as percentages of the label claim. The results were expressed as g gallic acid equivalents·mg·100 g^−1^ DW. For each treatment, three biological replicates were performed.

### 4.3. Total Proanthocyanidin (PAC) Analysis

#### 4.3.1. p-Dimethylaminocinnamaldehyde (DMACA) Method

The same materials used for polyphenol analysis were used for the analysis of total PAC content. The DMACA method [23] was used for total PAC content. Briefly, 0.025 g of freeze-dried sample was dissolved in 5 mL of milli-Q water. After heating at 95 °C for 15 min, the solution was incubated in cold water for 5 min and then filtered through a disposable syringe and a filter (0.22 µm). The samples and standard solutions were diluted appropriately in a microplate, mixed with 0.1% DMACA (FUJIFILM Wako Pure Chemical Corp., Osaka, Japan), and allowed to stand for 20 min, after which the absorbance at 640 nm was measured using a microplate reader (Bio Tek Epoch 2, Agilent, CA, USA). 1N HCl in methanol was used as a blank. Catechin (Funakoshi Co., Ltd.) was dissolved in methanol and used as the standard solution. For each treatment, three biological replicates were performed.

#### 4.3.2. Butanol/HCl Method

The butanol/HCl method [32] was also used to determine total PAC content. The solution was extracted using the same procedures as for the DMACA method. After 100 μL of extraction solution or standard solution was diluted with butanol, it was mixed with 25 μL of 2% (*w*/*v*) NH_4_Fe(SO_4_)_2_·12H_2_O (in 2MHCl) and 875 μL of butanol-conc. HCl (95:5). A portion of the mixture was placed in a screw-capped test tube and the remainder was used as a blank. The mixture was heated at 105 °C for 30 min, with stirring every 10 min, and then cooled in cold water for 15 min, after which the absorbance at 550 nm was measured. Procyanidin B_2_ (FUJIFILM Wako Pure Chemical Corp., Osaka, Japan) was dissolved in methanol and used as the standard solution. For each treatment, three biological replicates were performed. 

### 4.4. Mean Degrees of Polymerization (mDP) of PACs Polymerization

The mDP of PAC was estimated using the method of Sugamoto et al. [13]. The structural formula of the PAC in rabbiteye blueberry is shown in Figure 1 based on a report by Matsuo et al. [10]. Briefly, about 200 mg of freeze-dried sample was weighed, 40 mL of ultrapure water was added, and the mixture was incubated at 95 °C for 15 min then left on ice for 5 min. The solution was then filtered through a filter (0.20 µm), transferred to a flask, frozen in liquid nitrogen, and dried in a freeze-dryer (FDU-2100, EYELA, Tokyo, Japan). Then, 2.5 mg of freeze-dried powder was dissolved thoroughly in 500 mL of ethanol solution consisting of 5% (*v*/*v*) 2-mercaptoethanol, 4% (*v*/*v*) 0.5 M HCl, and 32% (*v*/*v*) H_2_O. The mixture was then heated at 70 °C for 7 h. The resulting thiolysis products were filtered according to the usual method and the filtrate was analyzed using a HPLC system. A Q-Exactive mass spectrometry system (Thermo Fisher Scientific, Waltham, MA, USA) equipped with a UV–visible detector and an ODS Hypersil C18 column (4.6 mm × 250 mm, 5 mm, Thermo Fisher Scientific, USA) was used for detailed analysis. The separation conditions were as follows: flow rate, 0.8 mL/min; elution solvents, A (0.1% formic acid in water) and B (acetonitrile); and gradient program, 10–28% B from 0 to 30 min and 28–82% B from 30 to 40 min. UV detection was analyzed at a wavelength of 280 nm. Three biological replicates were performed for each treatment. The mDP of PAC was calculated as follows: mDP = [sum of (2-hydroxyethylthio adducts × n) + sum of (free flavan-3-ol × n)]/[total free flavan-3-ol], where n is the degree of polymerization of the detected flavan-3-ol based on thiolysis. Average percentage of A-type bonds = [sum of (thiolysis compounds containing A-type bonds × n)/total free flavan-3-ol]/mDP × 100. Average percentage of cinchonain I units = [sum of (thiolysis compounds containing cinchonain I units × n)/total free flavan-3-ol]/mDP × 100.

### 4.5. Antioxidant Activity

The radical scavenging activity based on DPPH was measured through a partially modified microplate method [33]. About 0.05 g of freeze-dried leaf or branch powder was weighed, 10 mL of 80% ethanol was added and stirred for 10 min, and the solution was filtered through a disposable syringe and a filter (0.2 µm) to prepare the extraction sample. To the extracted sample, an appropriate amount of 200 mM MES and 20% ethanol was added to 800 µM DPPH reagent (Tokyo Kasei Kogyo Co., Ltd., Tokyo, Japan). These samples were incubated at room temperature for 20 min and then analyzed as absorbance at 520 nm using a microplate reader (EPOCH-2, Product No.: EPOCH2NS-SN; Agilent Technologies, Santa Clara, CA, USA). Measurements were repeated in triplicate, and the amount of Trolox equivalent per 100 g of fresh fruit was calculated.

### 4.6. Statistical Analysis

All experimental data were obtained from measurements in triplicate (three extracts from three independent plants, three measurements per extract), and data in the tables and figures are presented as means ± standard deviation (*n* = 3). These results were evaluated for statistical significance at *p* < 0.05 using univariate analysis of variance (ANOVA) and Tukey’s post-hoc test.

## 5. Conclusions

The present study revealed that total polyphenol content, total PAC content, and their involved antioxidant activity in the leaves and branches of rabbiteye blueberries varied depending on harvest time and organ. We also revealed seasonal changes in the mDP of PACs in the leaves and branches of rabbiteye blueberry, and this finding is the first report of its kind to our knowledge. Since differences in the mDP of PACs may affect their bioactivity, such as their absorption in the body, virus suppression, and antioxidant capacity, it is important to select the harvest time and organs based on the intended application. In the future, we aim to expand the possibilities of new functional foods by utilizing the differences in total PAC content and their mDP.

## Data Availability

The raw data used in this study and its analyzed data sets are available from the corresponding researcher upon reasonable request.

## Supplementary Materials

The following supporting information can be downloaded at https://www.mdpi.com/article/10.3390/plants13131864/s1, Figure S1: Chromatograms of polyphenols extracted from leaf of ‘Kunisato 35 gou’ in April, Figure S2: Chromatogram of reaction products of proanthocyanidins using HPLC/MS of rabbiteye blueberry leaves.
