# Supplementary material for: Seasonal Variation and Mean Degree of Polymerization of Proanthocyanidin in Leaves and Branches of Rabbiteye Blueberry (Vaccinium virgatum Aiton)
Source: Plants (Basel). 2024 Jul 5;13(13):1864. doi: 10.3390/plants13131864 (PMC11244326; doi:10.3390/plants13131864)
Supplement: Supplementary file 1 [file plants-13-01864-s001.zip › plants-3078663-supplementary.pdf]

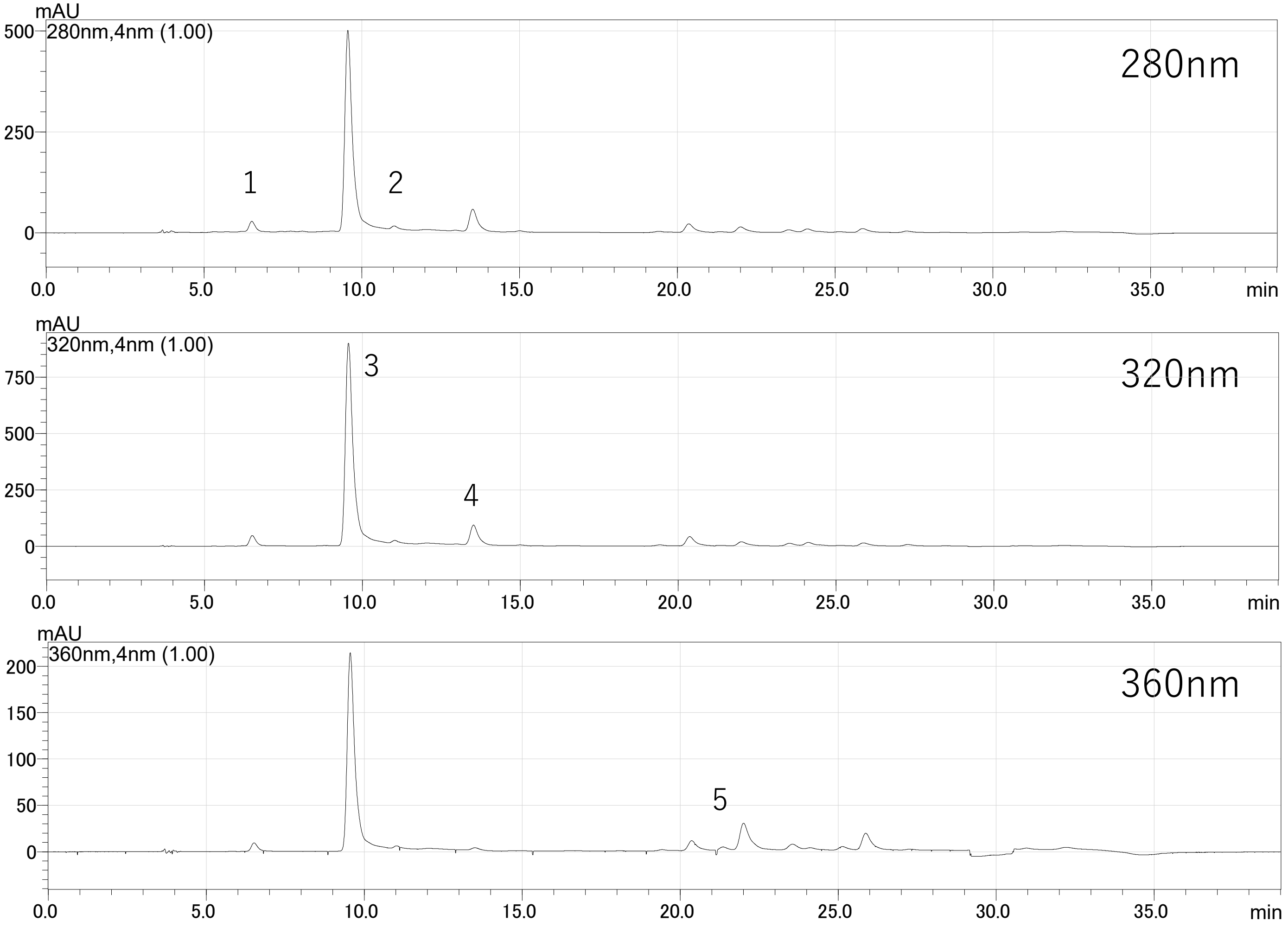

Figure S1. Chromatograms of polyphenols extracted from leaf of 'Kunisato 35 gou' in April: (1) catechin, (2) epicatechin, (3) chlorogenic acid, (4) caffeic acid, and (5) rutin.

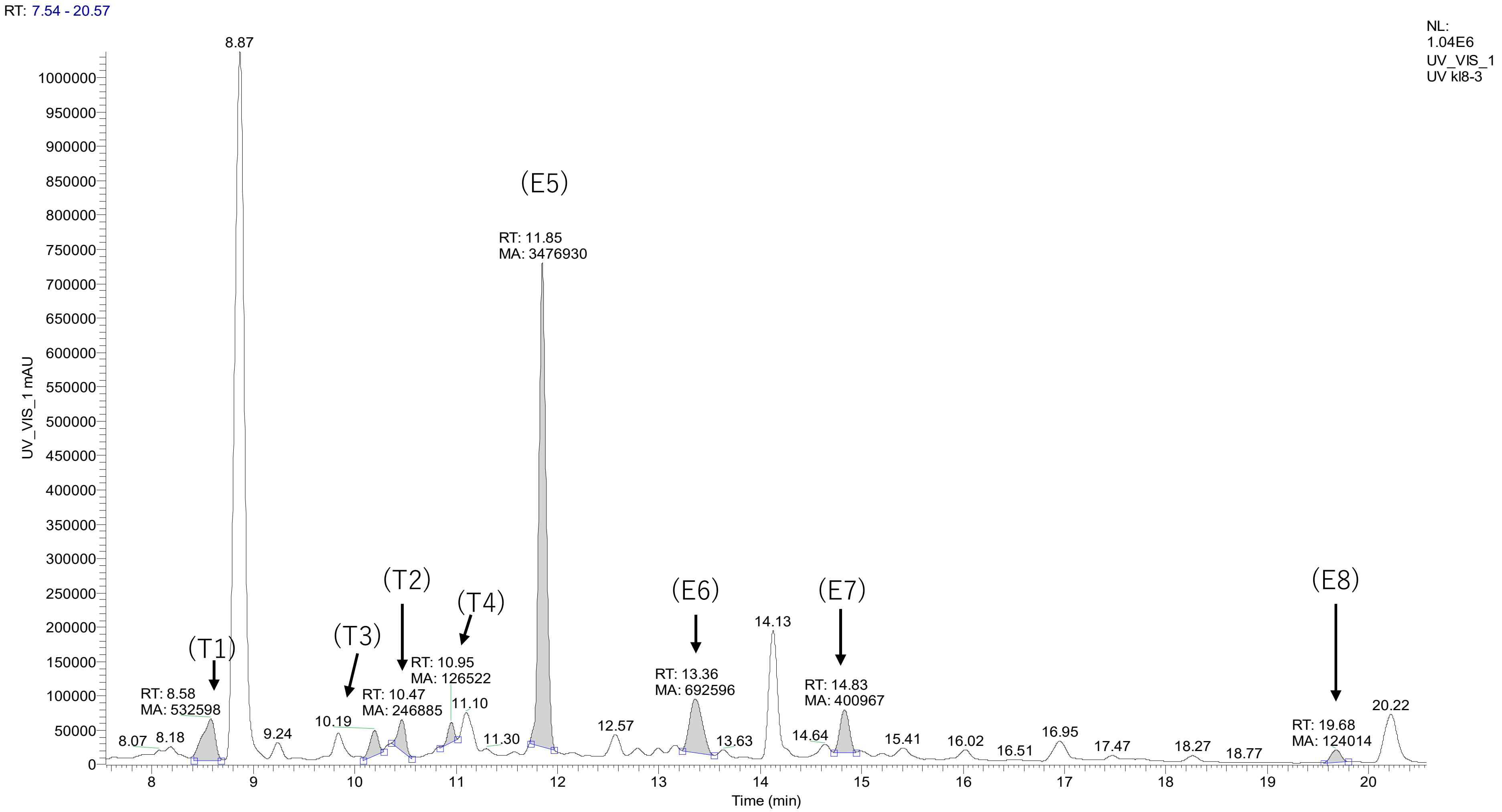

Figure S2. Chromatogram of reaction products of proanthocyanidins using HPLC/MS of rabbiteye blueberry leaves. T1-T4 in the figure represent terminal units of proanthocyanidin reaction products and E5-8 represent extension units.
